# Investigation for Factors Affecting Body Perception Disturbance in Patients with Low Back Pain by Mechanism-Based Classification of Pain: A Cross-Sectional Study

**DOI:** 10.1155/2023/5083084

**Published:** 2023-11-02

**Authors:** Yoshito Kurashima, Takumi Nakamura, Taishi Mukaiyama, Kenji Hasegawa, Hironobu Kuruma

**Affiliations:** ^1^Tokyo Spine Hospital, Tokyo, Japan; ^2^Department of Physical Therapy Sciences, Tokyo Metropolitan University, Tokyo, Japan; ^3^Sanraku Hospital, Tokyo, Japan; ^4^Arakawa Orthopaedic Rehabilitation Clinic, Tokyo, Japan

## Abstract

**Background:**

Central sensitization is a pathophysiological cause of chronic low back pain and is linked with psychosocial factors. The association between central sensitization (CS) and body perception disturbance is currently unclear, and no prior studies have investigated this relationship in patients with acute or subacute low back pain. The objective of this study was to investigate potential factors that influence body perception disturbance using a mechanistic classification of low back pain.

**Methods:**

This cross-sectional study was conducted at the time of initial physical therapy in patients with low back pain. During the study period, 169 patients were recruited. Pain intensity, disease duration, disability, CS, and body perception disturbance were evaluated. Patients were divided into three groups according to the pathology of low back pain, and multivariate analysis was used to examine factors affecting body perception disturbance. The dependent variable was Fremantle Back Awareness Questionnaire (FreBAQ); the independent variables were age, gender, BMI, VAS, disease duration, RDQ, and CS Inventory-9 (CSI-9).

**Results:**

A total of 117 patients were included in our analysis. According to the mechanistic classification of pain, 66 (56.4%), 36 (30.8%), and 15 (12.8%) patients were categorized as having nociceptive pain (NP), peripheral neuropathic pain (PNP), and CS pain (CSP), respectively. Patients with PNP or CSP were significantly older than those with NP (*p*  <  0.01). FreBAQ and RDQ scores were significantly higher in patients with CSP than those with NP (*p*  <  0.05). The results of multiple regression analyses indicated that CSI-9 scores were significantly associated with FreBAQ (*p*  <  0.01).

**Conclusion:**

Patients with CS syndrome and low back pain tend to have higher CSI-9 scores and be older. Body perception disturbance is influenced by CS or CS syndrome, regardless of the stage of low back pain, suggesting that patients with chronic low back pain tend to have low body image.

## 1. Introduction

Low back pain (LBP) is associated with a high physical and economic burden [1]. The lifetime prevalence of LBP is estimated to be as high as 80% [2], and recent studies have documented a consistent increase in its annual incidence; this is problematic, as there are few effective treatments for this condition [3, 4]. The lack of effective treatments may be attributed to the fact that most therapeutic strategies are based on biomedical models, which are reliant on anatomical and biomechanical factors [5, 6]. In contrast, the bio-psycho-social model provides a more comprehensive view of LBP by accounting for interactions with physical, psychological, and social influences.

Central sensitization (CS) has been recently reported to be involved in the pathophysiology of chronic pain [7, 8]. It is defined as a neurophysiological state caused by hyperexcitability of the central nervous system, and CS syndrome (CSS) has been proposed as a comprehensive disease concept in which CS is involved [9]. In terms of its association with LBP, CS has been reported to be associated with both psychosocial and cognitive-behavioral factors [10]. For example, CS in the acute phase of LBP may be a precursor for the transition to chronic LBP when it is combined with other psychological factors [11]. Furthermore, CS has been shown to mediate the relationship between pain intensity and psychosocial factors [12].

Previous studies have described the concept of body perception disturbance (BPD) as a disease-specific factor involved in the chronicity of LBP [13]. Body perception is the ability to accurately perceive and recognize one's own body parts and movements. Wand et al. developed the Fremantle Back Awareness Questionnaire (FreBAQ) to assess BPD in patients with chronic LBP and confirmed its reliability and validity [13]. While they demonstrated that BPD is associated with chronic LBP, their study only included patients with chronic LBP, as opposed to acute or subacute LBP. There is no previous study investigating the relationship between acute or subacute LBP and BPD, so the relationship between CS and BPD is currently unclear. These previous studies suggested that BPD may have an impact on the chronicity of LBP, so we hypothesized that BPD and CS are related and patients with CSS may have significantly higher BPD. This study is the first study to examine BPD in acute or subacute LBP.

Therefore, the purpose of this study was to examine factors that potentially influence body perception disturbance in patients with LBP, based on differences in the mechanistic classification of pain.

## 2. Methods

### 2.1. Study Design

This cross-sectional study was conducted with approval from the ethics committee of Tokyo Metropolitan University Arakawa (approval number: 20071).

### 2.2. Participants

This study included 121 patients who received physical therapy after medical consultation at our clinic, between March and November 2021. Patients were included if they were 20–65 years old and able to provide valid responses to the administered questionnaires. LBP was defined in accordance with the criteria proposed by the Japanese medical guidelines for LBP in 2019 [10]: “pain which is located in the back side of the trunk between 12 rib and gluteal folds, lasts for at least one day, with or without unilateral or bilateral radiating pain in lower limb.” Exclusion criteria consisted of the following: paralysis, tumor, infection, fresh vertebral fracture, pregnancy, those who are attending psychosomatic medicine or psychiatry, and indemnification problem.

### 2.3. Procedure (Figure 1)

The following parameters were extracted from the clinic medical records: age, gender, height, weight, body mass index (BMI), pain intensity, disease duration, FreBAQ, CSI-9, and Roland–Morris Disability Questionnaire (RDQ). All data were recorded during the first physical therapy session.

#### 2.3.1. Classification of Pain Mechanism

The classification of pain based on its underlying mechanism was determined by the physical therapist who was in charge of the first physical therapy session for each patient. The method of classification conformed with that of Nijs et al. [14] who used the following diagnostic criteria: (1) “is neuropathic pain present and able to explain the clinical picture?”; (2) “is there a disproportionate pain experience?”; (3) “is there a diffuse pain distribution?”; and (4) “is the CSI-9 score ≥20?”.

In addition, the physical therapist accounted for physical examination results (e.g., medical interviews and neurological tests) and classified pain into three groups (nociceptive pain (NP), peripheral neuropathic pain (PNP), and CS pain (CSP)). Mixed or uncertain pain types were excluded. A preliminary study was conducted among 10 physical therapists to evaluate the inter-rater reliability of this classification method; the Fleiss' kappa value was 0.638, indicating substantial agreement (Table 1).

### 2.4. Outcome Measurements

The primary outcomes were the Japanese versions of FreBAQ and CSI-9.

The following secondary outcomes were evaluated via a questionnaire survey that was administered during the initial physical therapy session: basic patient characteristics, visual analogue scale (VAS), disease duration, FreBAQ, CSI-9, and Japanese version of RDQ. Basic patient characteristics and disease duration were obtained from the medical records.

Basic patient characteristics included age, gender, height, weight, and BMI. Disease duration was defined as the number of days elapsed from the date of pain onset to the date of the first physical therapy evaluation.

The VAS [15] was used to measure pain intensity, which ranged from 0 (no pain) to 100 (maximum pain imaginable). Body perception disturbance was evaluated using the Japanese version of the FreBAQ [16], which was developed by Nishigami et al. to assess body perception disturbance; its validity and reliability have been previously confirmed [13, 17]. The FreBAQ is a questionnaire that can assess body perception disturbance and consists of a total of 9 questions to subfactors of neglect-like symptoms, proprioception, and body image. The questions are answered on a 5-point scale from 0 to 4, with higher values indicating greater body perception disturbance. Although no cutoff values have been reported, correlation with pain intensity, disability, depression, and pain catastrophizing has been reported. The CSI-9 is a questionnaire used to screen for CSS and has been reported to have high validity and reliability [18]. The questionnaire consists of 9 questions with a 5-point scale from 0 to 4, with a cutoff score of 20 or higher is considered suspicious for CS syndrome (10–19: mild and 20 or higher: moderate/severe). The RDQ [19] was used to evaluate impairments in activities of daily living due to LBP. Scores range from 0 to 24 points, with higher scores reflecting greater impairment.

### 2.5. Sample Size Calculation

The target sample size was for 180 participants in our research period with an effect size as *d* = 0.21 by referencing a previous study [20] which investigated the relationship between pain intensity and its mechanism.

### 2.6. Statistical Analysis

The Shapiro–Wilk test was used to examine the distributional normality of a sample, and FreBAQ and CSI-9 were consistent with a normal distribution, but the other was not. The one-way analysis of variance (ANOVA) or Kruskal–Wallis test was used to examine differences among groups for demographic data, VAS, FreBAQ, CSI-9, and RDQ. The *χ*^2^ test was used for gender. Multiple comparisons and Tukey's test were conducted as post hoc tests. The independent variable was the classification (group) of pain; the dependent variables were age, height, weight, gender, BMI, VAS, disease duration, FreBAQ, RDQ, and CSI-9. In addition, the one-way ANOVA was used to examine differences among groups for disease stage. The independent variable was disease stage (acute <4 weeks, subacute: 4 weeks-3 months, and chronic ≧3 months); the dependent variable was FreBAQ.

A multiple regression analysis (using the forced entry method) was conducted to examine factors that potentially influenced body perception disturbance. The dependent variable was FreBAQ; the independent variables were age, gender, BMI, VAS, disease duration, RDQ, and CSI-9.

All statistical analyses were performed using IBM SPSS Statistics version 26.0 (IBM SPSS Statistics, Version 26.0; IBM Corp., Armonk, NY, U.S.A.). The level of statistical significance was set at 0.05.

## 3. Results

**Figure 1:**
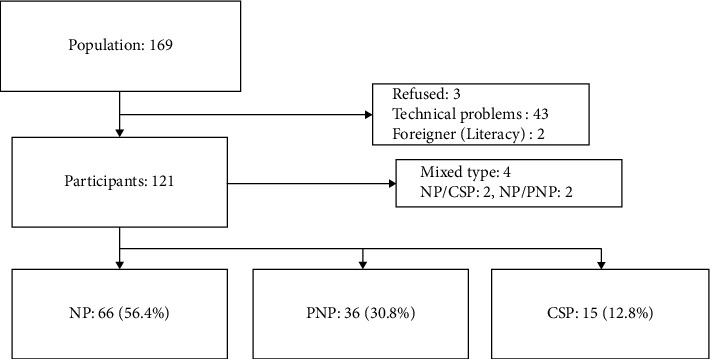
Flow diagram.

Figure 1 shows the study flow and the number of subjects excluded due to missing data at each stage of the analysis and the reasons for this exclusion. During the study period, 169 patients with LBP were recruited. Fifty-two patients were excluded due to the following reasons: did not provide informed consent (*n* = 3); unable to complete the assessment due to technical problems (*n* = 43); and mixed pain type (*n* = 4). No patients were classified with an uncertain pain type. Therefore, a total of 117 patients were included in our analysis.

According to the mechanistic classification of pain determined during the first physical therapy session, 66 (56.4%), 36 (30.8%), and 15 (12.8%) patients were categorized as having NP, PNP, and CSP, respectively.

Patient characteristics and outcomes according to pain type are summarized in Table 2. Patients with PNP or CSP were significantly older than those with NP (*p* ≤ 0.001). Gender, height, weight, and BMI were not significantly different among pain groups (*p* = 0.549, 0.918, 0.496, 0.348) (Table 2). To clarify the influence of age, a parallelism test was conducted with age as a covariate. While all outcomes were parallel, this did not reach statistical significance. Thus, one-way ANOVA and Kruskal–Wallis tests were conducted to examine differences among pain groups.

Comparisons of outcome measures among pain groups are shown in Tables 2–5. The results of the one-way ANOVA indicated that CSI-9 scores were significantly higher in patients with CSP than those with NP or PNP (*p* ≤ 0.001) (Tables 4 and 5). Also, FreBAQ and RDQ scores were significantly higher in patients with CSP than those with NP (*p* = 0.043, 0.034) (Tables 2–5). There were no significant intergroup differences in disease duration and VAS (*p* = 0.546, 0.214) (Table 2). Additionally, one-way ANOVA was also conducted to examine differences among groups divided by disease stages. However, there is no significant difference in FreBAQ (*p* = 0.496) (Table 6).

The results of the multiple regression analysis using the forced entry method are summarized in Table 7. CSI-9 was a significant independent predictor of FreBAQ (*p* ≤ 0.001). The standardized partial regression coefficient, which indicates the degree of influence from independent variables, was 0.409 for CSI-9. The rate of contribution from the regression formula (*R*^2^) was 0.280. The variance inflation factor was <2 for all independent variables.

## 4. Discussion

### 4.1. Mechanistic Classification of Pain

According to a classification of pain based on its underlying mechanism, 66 (56.4%), 36 (30.8%), and 15 (12.8%) patients had NP, PNP, and CSP, respectively. These results were generally similar to those reported by Smart et al. [20] (NP, 55%; PNP, 22%; CSP, 22%); however, the proportion of patients with CSP in the present study was slightly lower. This distribution in pain type may be attributed to the pathology of the pain and disease duration. Chronic pain is generally defined as pain persisting for >3 months [21]. CS is considered to be a form of chronic pain that becomes more prominent with time. However, in practice, the diagnosis of chronic pain and CS is not only based on the disease stage but also on the integration of various findings. In a study conducted among patients with musculoskeletal disorders, Tanaka et al. [22] reported that 15.17% of patients had mild CS (CSI score of 30–39) and 11.00% had moderate-to-severe CS (CSI score of >40). The number of patients with CS was slightly lower than that in our study. However, their study was only assessed CSI-9 scores, and the actual number of patients with CS and CSS was expected to be different. Therefore, based on these previous studies, we believe that the results of the present study are externally valid for patients with LBP.

### 4.2. Comparison of Basic Patients' Characteristics

Patients with PNP or CSP were significantly older than those with NP. The reason for this difference may be related to the pathophysiology of chronic pain. Apkarian et al. [23] reported that the dorsolateral prefrontal cortex is dysfunctional in patients with chronic LBP. Furthermore, aging causes a decline in function in this region. [24] Antonella et al. reported that elderly individuals have lower pain thresholds [25] and are more prone to CS. Thus, patients with CSP are more likely to be older and have a dysfunctional dorsolateral prefrontal cortex.

### 4.3. Comparison of Outcomes among Pain Groups

The significant differences in CSI-9 scores among the pain groups can be attributed to the fact that CSI-9 is used to screen for CS and CSS. The higher its scores, the more its pathology can be closer to CS. So, the results of CSI-9 are directly related to the classification results. This may be due to the inclusion and assessment of patients with LBP at their first physical therapy session. Chronic LBP is defined by the persistence of pain for at least 3 months, and CS becomes increasingly prominent over time. However, in the present study, patients were divided into groups based not only on disease duration alone but also by accounting for a range of physical findings; this may have explained the lack of difference in disease duration among groups. On the other hand, there was no significant difference in VAS scores among the groups. This finding differs from that of other studies [20]. Shigetoh et al. reported that CS mediates pain intensity and psychological factors [12]. Thus, the absence of a significant difference in pain intensity may be due to the low prevalence of psychological factors in some patients with CSP. In addition, the present study included patients with acute and subacute LBP; pain intensity and CS may not be related during these earlier stages of LBP. As current evidence indicates that pain intensity is associated with CS in patients with chronic LBP, it can be inferred that the strength of this association increases over the disease course.

### 4.4. Multiple Regression Analysis for Body Perception Disturbance

The multiple regression analysis indicated that CSI-9 was an independent and significant factor associated with BPD in patients with LBP. These results are consistent with those of previous studies on patients with chronic LBP. Thus, CS may be associated with BPD during the early acute and subacute phases of LBP, and this association becomes increasingly evident following the transition to the chronic stage. Nevertheless, the rate of contribution of CSI-9 was low (28.0%), suggesting the potential influence of other factors. LBP is a complex condition with a multifactorial etiology. A bio-psycho-social model has been previously proposed to account for the effects of psychological, social, and biophysical factors [4]. However, we did not evaluate outcomes related to psychological factors that are believed to be involved in pain chronicity [10, 11]. Therefore, although it was clear that CSS was associated with FreBAQ, the contribution of this factor was limited.

In the one-way ANOVA, FreBAQ scores were higher in patients with CSP than those with NP. Bogduk et al. [26] investigated the cause of LBP using nerve blocks and found that NP was the major contributor. Therefore, while NP may be predominant in the early stages of LBP, the mechanism of pain generation may change with chronicity. Psychosocial factors such as kinesiophobia and pain catastrophizing may be facilitators of CS [27] and CSP; they have also been shown to affect FreBAQ. In this study, there is no significant difference in FreBAQ among groups divided by disease stages. Furthermore, the contribution of CSI-9 was higher than that of disease duration in multiple regression analysis for FreBAQ. These results may indicate that the factors leading to chronicity are more influenced by body perception disturbance than by the disease duration itself. Thus, these factors may be used to predict chronicity [28].

There were some limitations in this study. First, we could not correct sufficient sample size. The target sample size was set for 180 participants in our research period with an effect size as *d* = 0.21 by referencing a previous study that investigated the relationship between pain intensity and its pain mechanism. Second, this study was a single-institutional study, so external validity was unclear. Third, this study was a cross-sectional study, which does not provide a causal association. We need to conduct further investigation about the chronicity of LBP or CS with a longitudinal study.

## 5. Conclusion

The results of this study suggest that patients with CS or CSS and low back pain tend to have higher CSI-9 scores and be older. Body perception disturbance is influenced by CS or CSS, regardless of the stage of low back pain, thus reflecting that patients with chronic low back pain tend to have low body image.

## Figures and Tables

**Table 1:** Cross tabulation of the results for mechanism-based classification of pain.

	Pt A	Pt B	Pt C	Pt D	Pt E
PT①	NP	NP	NP	NP	NP
PT②	NP	NP	NP	MP	NP
PT③	NP	NP	NP	NP	NP
PT④	NP	NP	NP	NP	NP
PT⑤	NP	NP	NP	NP	NP
PT⑥	NP	NP	NP	MP	NP
PT⑦	NP	NP	NP	NP	NP
PT⑧	NP	NP	NP	NP	NP
PT⑨	NP	NP	NP	NP	NP
PT⑩	NP	NP	NP	NP	NP

PT: physical therapist; Pt: patient; NP: nociceptive pain; MP: mixed pain.

**Table 2:** Demographic data in each group.

	NP (*n* = 66)	PNP (*n* = 36)	CSP (*n* = 15)	*d*	*r*	*p* values
Gender (M/F)	30/36	20/16	7/8	2		0.549
Age (years)	42.7 ± 10.3	48.7 ± 12.2	52.3 ± 6.6	2		≤0.001^*∗*^
Height (cm)	163.8 ± 21.1	165.1 ± 7.4	164.0 ± 7.1	2		0.918
Weight (kg)	62.7 ± 13.7	64.1 ± 12.2	67.3 ± 13.4	2		0.496
BMI (kg/m^2^)	22.7 ± 3.3	23.4 ± 3.7	24.9 ± 4.4	2		0.348
Duration (days)	151.9 ± 485.8	34.8 ± 38.6	249.9 ± 485.6	2	0.11	0.546
VAS (cm)	5.0 ± 2.4	5.4 ± 2.3	6.0 ± 1.9	2	0.26	0.214
RDQ	6.9 ± 5.4	8.8 ± 5.3	9.7 ± 4.8	2	0.46	0.040^*∗*^

Mean ± standard deviation (SD). BMI: body mass index; VAS: visual analogue scale; RDQ: Roland-Morris disability questionnaire NP: nociceptive pain; PNP: peripheral neuropathic pain; CSP: central sensitization pain. ^*∗*^: Statistically significant.

**Table 3:** Outcome measurements in each group.

	NP (*n* = 66)	PNP (*n* = 36)	CSP (*n* = 15)
VAS (cm)	5.0 ± 2.4	5.4 ± 2.3	6.0 ± 1.9
Duration (days)	151.9 ± 485.8	34.8 ± 38.6	249.9 ± 485.6
FreBAQ	10.6 ± 5.7	11.2 ± 6.7	14.9 ± 5.5
CSI-9	14.0 ± 5.7	13.8 ± 7.0	24.8 ± 3.5
RDQ	6.9 ± 5.4	8.8 ± 5.3	9.7 ± 4.8

Mean ± SD. VAS: visual analogue scale; FreBAQ: fremantle back awareness questionnaire; CSI-9: central sensitization inventory-9; RDQ: Roland-Morris disability questionnaire. NP: nociceptive pain; PNP: peripheral neuropathic pain; CSP: central sensitization pain.

**Table 4:** Difference among groups by one-way ANOVA for mechanism-based classification.

	*F* value	*p* values	*η* ^2^
FreBAQ	2.974	0.049^*∗*^	0.05
CSI-9	22.024	≤0.001^*∗*^	0.28

FreBAQ: fremantle back awareness questionnaire; CSI-9: central sensitization inventory-9. ^*∗*^: Statistically significant.

**Table 5:** The result of post hoc analysis for age, FreBAQ, CSI-9, and RDQ.

	NP	PNP	CSP
*Age*			
NP	1.000	0.012^*∗*^	0.003^*∗*^
PNP		1.000	0.337
CSP			1.000

*FreBAQ*			
NP	1.000	0.996	0.043^*∗*^
PNP		1.000	0.080
CSP			1.000

*CSI-9*			
NP	1.000	0.958	≤0.001^*∗*^
PNP		1.000	≤0.001^*∗*^
CSP			1.000

*RDQ*			
NP	1.000	0.060	0.034^*∗*^
PNP		1.000	0.531
CSP			1.000

FreBAQ: fremantle back awareness questionnaire; CSI-9: central sensitization inventory-9; RDQ: Roland-Morris disability questionnaire. NP: nociceptive pain; PNP: peripheral neuropathic pain; CSP: central sensitization pain. ^*∗*^: Statistically significant.

**Table 6:** Difference in FreBAQ among groups by one-way ANOVA for disease stages.

	*F* value	*p* values	*η* ^2^
FreBAQ	0.671	0.496	2.01

FreBAQ: fremantle back awareness questionnaire.

**Table 7:** The result of multiple regression analysis for FreBAQ.

Variable	*β*	Single correlation	*p* values
CSI-9	0.409	0.467	≤0.001^*∗*^
RDQ	0.188	0.188	0.059
VAS	0.103	0.103	0.272
Disease duration	0.035	0.035	0.692
BMI	−0.038	−0.06	0.676
Age	−0.06	−0.006	0.515
Gender	−0.104	−0.104	0.271
*R* ^2^	0.280^∗^		

CSI-9: central sensitization inventory-9; RDQ: roland-morris disability questionnaire; VAS: visual analogue scale; BMI: body mass index. ^*∗*^: Statistically significant.

## Data Availability

The data that support the findings of this study are openly available in Miyakodori at https://tokyo-metro-u.repo.nii.ac.jp/.
